# Three Large Language Models (LLMs), One Heart: A Comparative Evaluation of ChatGPT, Claude, and DeepSeek in Cardiac Imaging Patient Education

**DOI:** 10.7759/cureus.115285

**Published:** 2026-08-27

**Authors:** Tooba Fatima Iram, Haroon Abdullah, Naazira Begum, Suraiya Begum, Shaheed Antlee

**Affiliations:** 1 Cardiac Critical Care, CARE Hospitals, Hyderabad, IND; 2 Emergency Medicine, Mythri Hospitals, Hyderabad, IND; 3 Pathology, King Salman Bin Abdulaziz Medical City, Madinah, SAU; 4 Dermatology, King Salman Bin Abdulaziz Medical City, Madinah, SAU

**Keywords:** artificial intelligence in radiology, cardiac imaging modalities, cardiac imaging-mri, health information literacy, large language models (llms), patient education material

## Abstract

Introduction

Clear, accurate, and accessible patient education is central to informed decision-making and high-quality cardiovascular care. Large language models (LLMs) are increasingly being used to generate health information, offering the potential to rapidly produce patient education materials. However, concerns remain regarding the readability, quality, clinical accuracy, and adherence to evidence-based recommendations of AI-generated content. This study compared the performance of ChatGPT (OpenAI, San Francisco, CA), Claude (Anthropic PBC, San Francisco, CA), and DeepSeek (DeepSeek Artificial Intelligence Co., Ltd., Hangzhou, China) in generating patient education leaflets for three commonly performed cardiac imaging procedures.

Methodology

A cross-sectional study was conducted using standardized prompts to generate patient education leaflets for cardiac magnetic resonance imaging (CMR), coronary computed tomography angiography (CTCA), and invasive coronary angiography (ICA) using ChatGPT, Claude, and DeepSeek. Nine leaflets were evaluated for readability using Flesch-Kincaid Grade Level, Gunning Fog Index, Simple Measure of Gobbledygook (SMOG), Flesch Reading Ease, word count, and sentence count. Information quality was assessed using the modified DISCERN (mDISCERN) instrument. Guideline adherence was assessed using investigator-developed checklists based on recommendations from relevant professional societies and patient education resources. Expert clinical assessment was independently performed by two reviewers using a structured 16-point scoring system. Comparisons among the three models were performed using the Kruskal-Wallis test.

Results

DeepSeek demonstrated the most favorable overall readability profile, with the highest Flesch Reading Ease score (70.73 ± 1.74) compared with ChatGPT (50.70 ± 7.88) and Claude (60.93 ± 5.51; H(2) = 7.20, p = 0.027). DeepSeek achieved the highest mean mDISCERN score (3.83 ± 0.29), followed by ChatGPT (3.50 ± 0.50) and Claude (2.67 ± 0.29), although the difference was not statistically significant (H(2) = 5.593, p = 0.061). ChatGPT demonstrated the highest mean guideline adherence (91.54 ± 3.39%), followed by DeepSeek (89.33 ± 3.41%) and Claude (88.07 ± 6.48%; H(2) = 0.707, p = 0.702). DeepSeek achieved the highest expert clinical assessment score (16.00 ± 0.00), followed by ChatGPT (15.67 ± 0.58) and Claude (14.33 ± 0.58), with no statistically significant difference (H(2) = 5.394, p = 0.067). A significant difference was also observed in total word count (H(2) = 7.20, p = 0.027).

Conclusions

All three LLMs generated high-quality patient education leaflets for common cardiac imaging procedures, with each model demonstrating distinct strengths across different evaluation domains. DeepSeek showed the most favorable readability and achieved the highest information quality and expert clinical assessment scores, whereas ChatGPT demonstrated the greatest guideline adherence. However, all three models produced content above recommended patient health-literacy standards. LLMs may therefore serve as valuable clinician-assisted tools for developing cardiac imaging education materials, but expert review and readability optimization remain essential before clinical implementation.

## Introduction

For many patients, the journey through cardiovascular care begins not with treatment, but with uncertainty. Questions regarding the purpose of an investigation, how it will be performed, and the associated risks are common sources of anxiety before cardiac imaging procedures. Clear, accurate, and accessible patient education is therefore fundamental to informed decision-making, improving health literacy, reducing procedural anxiety, and supporting patient-centered care [1]. However, developing and maintaining high-quality educational materials requires considerable clinical expertise, time, and regular updating to reflect evolving evidence and guideline recommendations.

Cardiac magnetic resonance imaging (CMR), coronary computed tomography angiography (CTCA), and invasive coronary angiography (ICA) are among the most frequently performed investigations in modern cardiovascular practice. Despite their widespread use, these procedures differ substantially in their indications, preparation requirements, complexity, and potential risks, making comprehensive and procedure-specific patient education essential. Furthermore, many patient information resources exceed the recommended reading level, potentially limiting comprehension among individuals with lower health literacy.

Recent advances in generative AI, particularly large language models (LLMs), have introduced a promising approach to rapidly generating patient education materials. Contemporary LLMs such as ChatGPT (OpenAI, San Francisco, CA), Claude (Anthropic PBC, San Francisco, CA), and DeepSeek (DeepSeek Artificial Intelligence Co., Ltd., Hangzhou, China) are capable of producing coherent and contextually relevant medical information within seconds and are increasingly being explored for applications in patient education, clinical documentation, and medical education [2-5]. Nevertheless, concerns remain regarding the accuracy, completeness, readability, and guideline adherence of AI-generated medical content, highlighting the need for rigorous evaluation before widespread clinical implementation.

Although several studies have assessed LLM-generated patient education across different medical specialties, most have focused on a single model or a single clinical topic. Comparative evaluations of multiple contemporary LLMs using standardized prompts and comprehensive assessment frameworks remain limited, particularly in cardiovascular imaging.

Therefore, this study compared ChatGPT, Claude, and DeepSeek in generating patient education leaflets for CMR, CTCA, and ICA using an identical standardized prompt. The generated leaflets were evaluated for readability, information quality using the modified DISCERN (mDISCERN) instrument, adherence to procedure-specific guideline recommendations, and overall expert clinical assessment. By comparing three leading LLMs across multiple cardiac imaging modalities, this study aims to provide evidence regarding their potential role in developing high-quality patient education materials for clinical practice.

## Materials and methods

Study design

A cross-sectional original research study was conducted in July 2026 to evaluate the performance of three LLMs - ChatGPT, Claude, and DeepSeek - in generating patient education leaflets for common cardiac imaging procedures. Since this study did not involve human participants, patient data, or identifiable personal information, ethical approval was not required.

Generation of patient education leaflets

Three commonly performed cardiac imaging procedures were selected: CMR, CTCA, and ICA. A standardized prompt was used to generate patient education leaflets for each procedure using all three LLMs. The prompt instructed each model to produce a leaflet for an adult patient written in plain English at approximately an eighth-grade reading level and to include the following sections: (1) What is the procedure? (2) Why is the procedure performed? (3) How should I prepare for the procedure? (4) What happens during the procedure? (5) What are the benefits? (6) What are the possible risks and complications? (7) What should I expect after the procedure? (8) Frequently asked questions. Each model received the identical prompt without further modification or regeneration of responses, resulting in a total of nine patient education leaflets.

The standardized prompts and the complete AI-generated patient education leaflets are provided in the Appendices to facilitate reproducibility and qualitative comparison of model outputs.

Outcome measures

All qualitative assessments were performed independently by two reviewers, and any disagreements were resolved through discussion until consensus was achieved.

Readability assessment

Readability was assessed using the WebFX Readability Test Tool [6]. The generated leaflets were analyzed for total word count, total sentence count, Flesch Reading Ease score, Flesch-Kincaid Grade Level, Gunning Fog Index, and Simple Measure of Gobbledygook (SMOG). The Flesch Reading Ease Score measures the ease of understanding of written text, with higher scores indicating greater readability, while the Flesch-Kincaid Grade Level estimates the U.S. school grade required to comprehend the text [7]. The Gunning Fog Index and SMOG estimate the years of formal education needed to understand the material, with lower scores indicating easier readability. These validated readability indices are widely used to assess the accessibility of patient education materials [8,9].

Information quality assessment

Information quality was assessed using the mDISCERN instrument, a validated tool for evaluating the quality and reliability of written health information [10]. The mDISCERN consists of five criteria: clarity, reliability, balance and bias, provision of additional sources of information, and acknowledgment of areas of uncertainty. Each criterion was scored as either 0 (absent) or 1 (present), resulting in a maximum score of 5, with higher scores indicating better information quality.

Guideline adherence assessment

Guideline adherence was assessed using investigator-developed checklists based on recommendations from recognized professional societies and patient education resources. The CTCA checklist was derived from guidance published by the American College of Radiology (ACR), Radiological Society of North America (RSNA) RadiologyInfo, and the American Heart Association (AHA). The ICA checklist was based on patient education resources from the Society for Cardiovascular Angiography and Interventions (SCAI) Seconds Count initiative and the AHA. The CMR checklist was developed using recommendations from the Society for Cardiovascular Magnetic Resonance (SCMR) and RSNA RadiologyInfo. Guideline adherence was expressed as the percentage of checklist items fulfilled. Checklist items are included in the Appendices as an accessible Google Drive (Google LLC, Mountain View, CA) link.

Expert clinical assessment

Overall clinical quality was assessed using a structured scoring rubric evaluating factual accuracy, completeness, clarity, organization, clinical relevance, and suitability for patient education. Each leaflet was assigned a maximum score of 16 points, with higher scores indicating better overall clinical quality.

Statistical analysis

The collected data were entered into Microsoft Excel (Microsoft Corporation, Redmond, WA) and analyzed using IBM SPSS Statistics version 29.0 (IBM Corp., Armonk, NY). Continuous variables are presented as mean ± standard deviation (SD). Comparisons among the three LLMs were performed using the Kruskal-Wallis test. A two-sided p-value of <0.05 was considered statistically significant.

## Results

A total of nine patient education leaflets were generated by ChatGPT, Claude, and DeepSeek for three cardiac imaging procedures (CMR, CTCA, and ICA). The leaflets were evaluated for readability, information quality, guideline adherence, and expert clinical assessment. The results are summarized below.

Readability analysis

The readability characteristics of the patient education leaflets generated by ChatGPT, Claude, and DeepSeek are summarized in Table 1. No statistically significant differences were observed for the Flesch-Kincaid Grade Level (H(2) = 5.60, p = 0.061), Gunning Fog Index (H(2) = 4.36, p = 0.113), SMOG (H(2) = 4.62, p = 0.099), or sentence count (H(2) = 2.17, p = 0.337). Significant differences were observed for Flesch Reading Ease (H(2) = 7.20, p = 0.027) and word count (H(2) = 7.20, p = 0.027).

**Table 1:** Readability characteristics of patient education leaflets generated by ChatGPT, Claude, and DeepSeek Comparison of readability characteristics of patient education leaflets generated by ChatGPT, Claude, and DeepSeek for cardiac magnetic resonance imaging (CMR), coronary computed tomography angiography (CTCA), and invasive coronary angiography (ICA). Values are presented as mean ± standard deviation (SD). Comparisons among the three models were performed using the Kruskal-Wallis test, with the corresponding H statistic (H(2)) and p-values reported.

Variable	ChatGPT (Mean ± SD)	DeepSeek (Mean ± SD)	Claude (Mean ± SD)	Kruskal-Wallis H(2)	p-value
Flesch-Kincaid Grade Level	9.80 ± 1.61	6.60 ± 0.56	8.93 ± 1.30	5.6	0.061
Gunning Fog Index	12.37 ± 1.91	9.07 ± 1.10	11.57 ± 1.53	4.36	0.113
Simple Measure of Gobbledygook	9.43 ± 1.50	7.20 ± 0.56	8.70 ± 1.11	4.62	0.099
Flesch Reading Ease	50.70 ± 7.88	70.73 ± 1.74	60.93 ± 5.51	7.2	0.027
Sentence Count	90.67 ± 31.50	118.33 ± 10.02	105.67 ± 6.81	2.17	0.337
Word Count	1252.33 ± 120.81	1514.33 ± 38.42	1767.33 ± 112.51	7.2	0.027

Information quality

**Figure 1:**
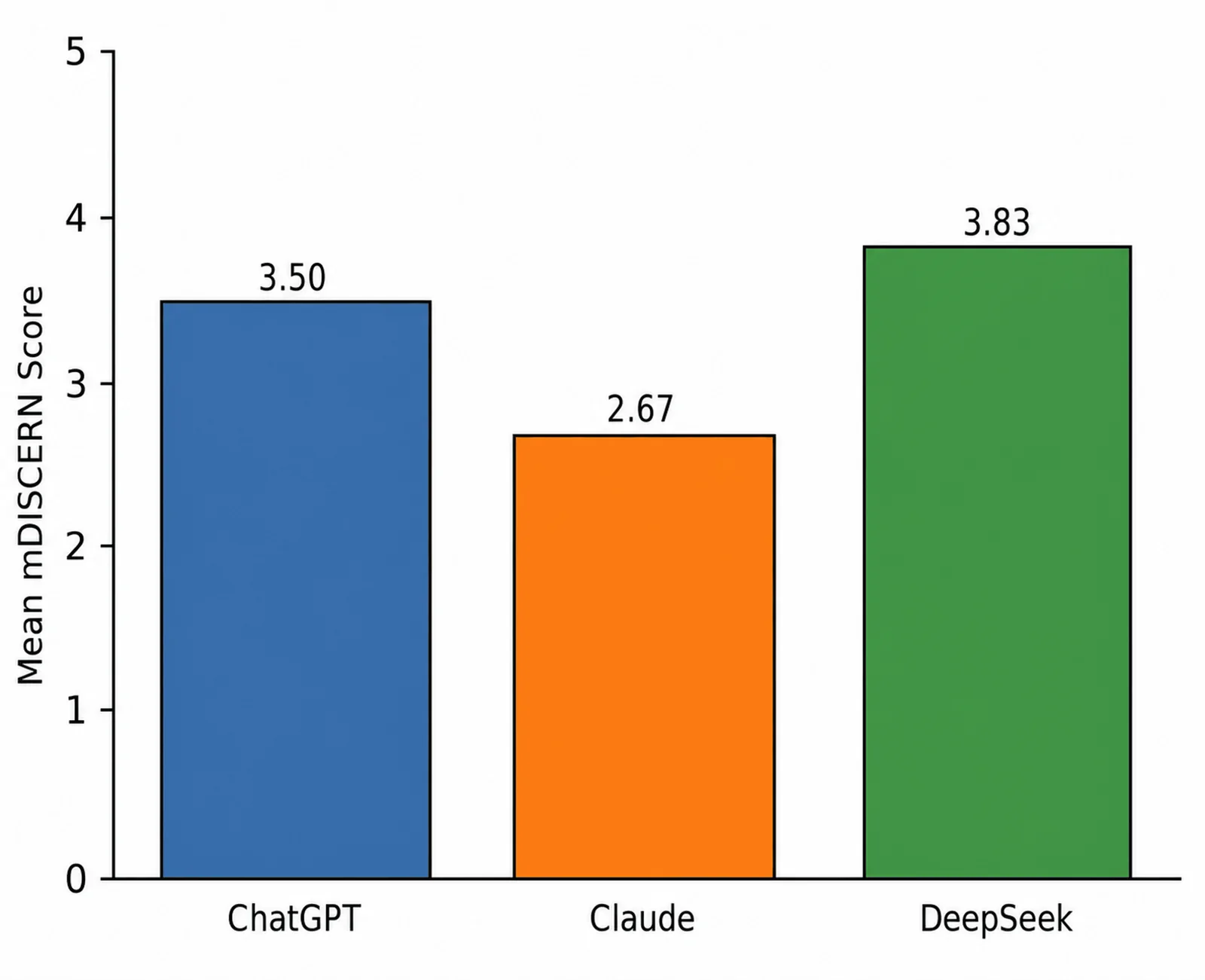
Mean mDISCERN score Comparison of the mean modified DISCERN (mDISCERN) scores for patient education leaflets generated by ChatGPT, Claude, and DeepSeek. Higher mDISCERN scores indicate better information quality and reliability of the generated patient education materials.

Information quality was evaluated using the mDISCERN instrument. DeepSeek achieved the highest mean mDISCERN score (3.83 ± 0.29), followed by ChatGPT (3.50 ± 0.50) and Claude (2.67 ± 0.29). Although DeepSeek obtained the highest overall information quality score, the differences among the three models were not statistically significant (Kruskal-Wallis H(2) = 5.593, p = 0.061) (Table 2, Figure 1).

**Table 2:** Information quality assessment using the modified DISCERN (mDISCERN) instrument Comparison of information quality scores of AI-generated patient education leaflets generated by ChatGPT, Claude, and DeepSeek for cardiac magnetic resonance imaging (CMR), coronary computed tomography angiography (CTCA), and invasive coronary angiography (ICA). Values are presented as mean ± standard deviation (SD). Higher mDISCERN scores indicate better information quality. Comparisons among the three models were performed using the Kruskal-Wallis test, with the corresponding H statistic (H(2)) and p-value reported. Statistical analysis: Comparisons among ChatGPT, Claude, and DeepSeek were performed using the Kruskal-Wallis test (H = 5.593, df = 2, p = 0.061).

Model	CMR	CTCA	ICA	Mean ± SD
ChatGPT	3.0	4.0	3.5	3.50 ± 0.50
DeepSeek	3.5	4.0	4.0	3.83 ± 0.29
Claude	3.0	2.5	2.5	2.67 ± 0.29
Kruskal-Wallis test	H(2) = 5.593	df = 2	p = 0.061	-

Guideline adherence

**Figure 2:**
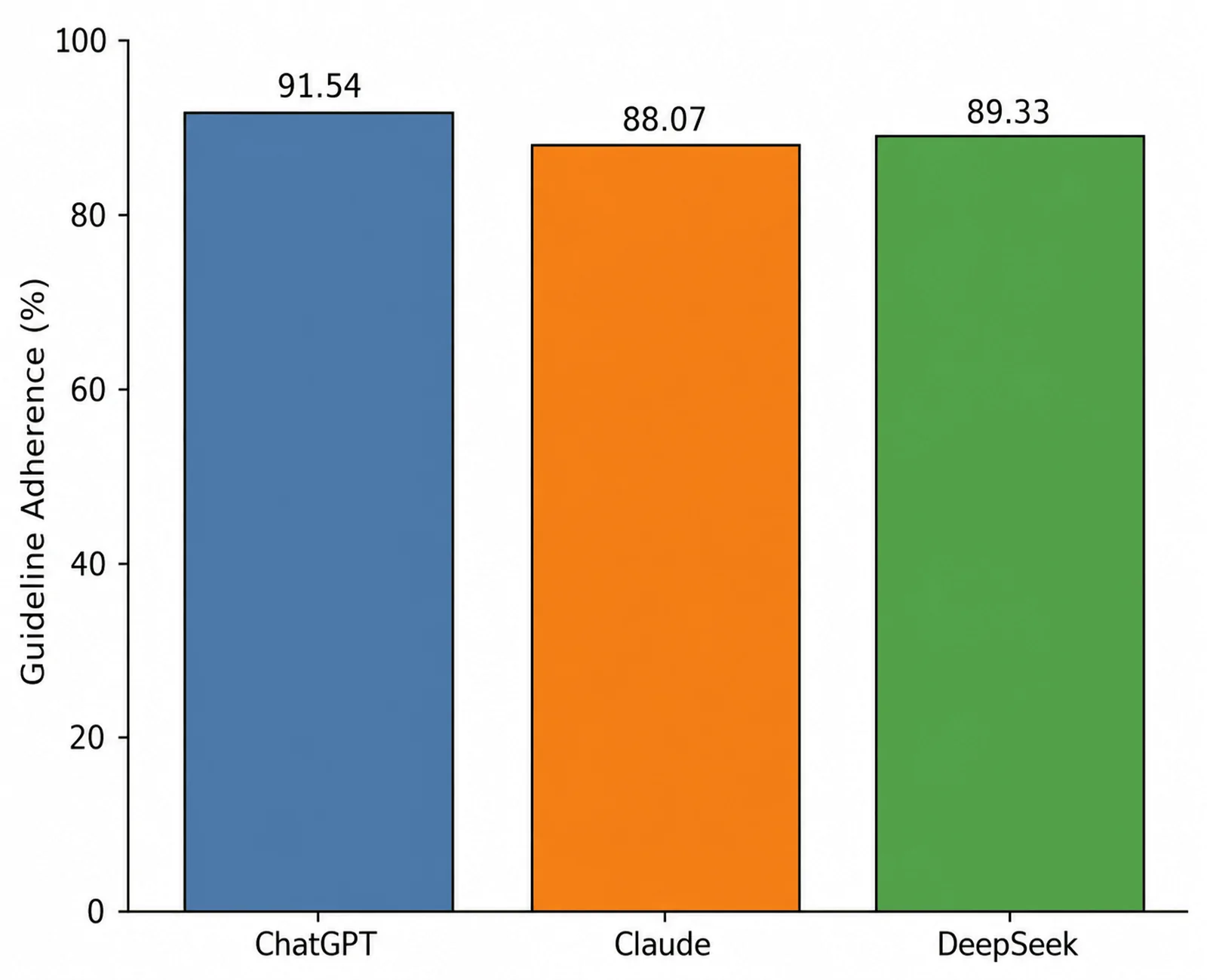
Mean guideline adherence (%) Comparison of the mean guideline adherence of patient education leaflets generated by ChatGPT, Claude, and DeepSeek. Values represent the mean percentage of checklist items fulfilled according to recommendations from professional society guidelines and patient education resources. Higher percentages indicate greater adherence to guideline-recommended content.

Guideline adherence scores are presented in Table 3. ChatGPT demonstrated the highest mean guideline adherence (91.54 ± 3.39%), followed by DeepSeek (89.33 ± 3.41%) and Claude (88.07 ± 6.48%). No statistically significant differences were observed among the three models (Kruskal-Wallis H(2) = 0.707, p = 0.702) (Table 3, Figure 2).

**Table 3:** Guideline adherence Comparison of guideline adherence of AI-generated patient education leaflets based on investigator-developed checklists derived from recommendations of recognized professional societies and patient education resources. Values are presented as mean ± standard deviation (SD). Statistical comparisons were performed using the Kruskal-Wallis test (H = 0.707, df = 2, p = 0.702). Abbreviations: AI, artificial intelligence; CMR, cardiac magnetic resonance imaging; CTCA, coronary computed tomography angiography; ICA, invasive coronary angiography; SD, standard deviation

Procedure	ChatGPT	Claude	DeepSeek
CMR	21.5/24 (89.6%)	20.5/24 (85.4%)	20.5/24 (85.4%)
CTCA	21.5/24 (89.6%)	20.0/24 (83.3%)	22.0/24 (91.7%)
ICA	21.0/24 (87.5%)	21.0/24 (87.5%)	20.0/24 (83.3%)
Mean ± SD (%)	91.54 ± 3.39	88.07 ± 6.48	89.33 ± 3.41
Kruskal-Wallis	H(2) = 0.707, p = 0.702	-	-

Expert clinical assessment

The expert clinical assessment scores are presented in Table 4. DeepSeek achieved the highest mean expert assessment score (16.00 ± 0.00), followed by ChatGPT (15.67 ± 0.58) and Claude (14.33 ± 0.58). Although DeepSeek achieved the highest overall expert assessment score, the differences among the three models were not statistically significant (Kruskal-Wallis H(2) = 5.394, p = 0.067).

**Table 4:** Expert clinical assessment Comparison of expert clinical assessment scores for patient education leaflets generated by ChatGPT, Claude, and DeepSeek for cardiac magnetic resonance imaging (CMR), coronary computed tomography angiography (CTCA), and invasive coronary angiography (ICA). Scores were assigned independently by two reviewers using a structured scoring rubric evaluating factual accuracy, completeness, clarity, organization, clinical relevance, and suitability for patient education. Values are presented as mean ± standard deviation (SD). Higher scores indicate better overall clinical quality. Comparisons among the three models were performed using the Kruskal-Wallis test, with the corresponding H statistic (H(2)) and p-value reported.

Procedure	ChatGPT	Claude	DeepSeek
CMR	16/16	14/16	16/16
CTCA	16/16	15/16	16/16
ICA	15/16	14/16	16/16
Mean ± SD	15.67 ± 0.58	14.33 ± 0.58	16.00 ± 0.00
Overall Comparison	H(2) = 5.394, p = 0.067	-	-

Overall performance

Across all evaluation domains, DeepSeek demonstrated the most favorable overall readability profile, achieved the highest mean mDISCERN score, and obtained the highest expert clinical assessment score, whereas ChatGPT demonstrated the highest mean guideline adherence. Statistically significant differences were observed only for Flesch Reading Ease and total word count, while no significant differences were identified for information quality (mDISCERN), guideline adherence, or expert clinical assessment.

DeepSeek consistently demonstrated the highest overall performance across multiple evaluation domains, whereas ChatGPT achieved the greatest adherence to guideline-recommended content. Claude produced clinically acceptable patient education leaflets but generally demonstrated lower information quality and guideline adherence than the other evaluated models.

## Discussion

AI is rapidly emerging as an important component of contemporary cardiovascular medicine, with applications spanning cardiovascular risk prediction, diagnosis, imaging, risk stratification, and personalized therapeutic management. Recent evidence highlights the growing potential of AI to support individualized cardiovascular care while also emphasizing the need for appropriate clinical validation and oversight [11,12]. 

The substantial strain placed on healthcare systems during the pandemic also demonstrated the need for scalable approaches to patient education and healthcare delivery when personnel and resources are limited. AI-based tools may therefore provide valuable support during future pandemics or healthcare crises by rapidly generating accessible patient education materials and reducing the informational burden on healthcare professionals, an application of AI in cardiology highlighted during the COVID-19 pandemic [13].

This cross-sectional study evaluated the performance of ChatGPT, Claude, and DeepSeek in generating patient education leaflets for three commonly performed cardiac imaging procedures: CMR, CTCA, and ICA. The generated leaflets were assessed for readability, information quality, guideline adherence, and expert clinical evaluation. Overall, all three LLMs produced clinically acceptable patient education materials with high information quality and strong adherence to professional guideline recommendations. While DeepSeek demonstrated superior readability and achieved the highest information quality and expert assessment scores, ChatGPT showed the greatest guideline adherence. However, no statistically significant differences were observed between the models for information quality, guideline adherence, or expert clinical assessment.

Cardiac imaging plays a central role in the diagnosis, risk stratification, and management of cardiovascular disease. Procedures such as CMR, CTCA, and ICA differ substantially in their indications, preparation, duration, and potential risks. Consequently, patients frequently experience anxiety and uncertainty before undergoing these investigations, particularly invasive procedures. High-quality patient education materials are therefore essential to improve patients' understanding of diagnostic procedures, reduce fear and anxiety, facilitate informed consent and shared decision-making, enhance health literacy, and improve the overall healthcare experience. However, the complexity of many cardiac imaging procedures can make them difficult for patients with limited health literacy to understand, highlighting the need for educational resources that are both clinically accurate and written in clear, accessible language [14,15].

The American Medical Association (AMA) and the U.S. Department of Health and Human Services recommend that patient education materials be written at approximately a fifth- to sixth-grade reading level to maximize comprehension among the general population [16]. In the present study, none of the three LLMs achieved this recommended standard. DeepSeek produced the highest Flesch Reading Ease score (70.73 ± 1.71), outperforming ChatGPT (50.70 ± 7.91) and Claude (60.93 ± 5.51). Similarly, DeepSeek generated the lowest Flesch-Kincaid Grade Level, Gunning Fog Index, and SMOG score, although these differences did not reach statistical significance. These findings suggest that DeepSeek may communicate complex medical information using simpler language while maintaining comparable clinical content. Nevertheless, all three models generated text above the recommended reading level for patient education, indicating that further simplification is required before routine clinical use. Similar findings have been reported by previous studies evaluating LLM-generated patient education materials, which demonstrated that although AI-generated content is generally understandable and accurate, it frequently exceeds recommended health literacy standards. Previous research has also shown that readability can be substantially improved through prompt engineering and iterative refinement of AI-generated text, suggesting that targeted prompting may enhance the accessibility of patient education materials without compromising informational quality [17].

Information quality was evaluated using the mDISCERN instrument, which is an updated version of the original DISCERN tool, tailored to assess the quality of written health information [18]. DeepSeek achieved the highest mean mDISCERN score, followed by ChatGPT and Claude, although the differences were not statistically significant. This suggests that all three LLMs were capable of generating educational material with acceptable reliability and balanced health information. Similar findings have been reported in previous studies evaluating LLM-generated patient education materials in other medical specialties, where AI-generated content generally demonstrated good quality but varied in completeness and presentation.

Guideline adherence was consistently high across all three models, with mean adherence exceeding 88%. ChatGPT demonstrated the highest overall adherence to recommendations derived from professional society guidelines, although the differences between models were small and not statistically significant. Likewise, expert clinical assessment showed high scores for all three LLMs, with DeepSeek receiving perfect expert scores across all three imaging procedures. These findings suggest that contemporary LLMs can successfully incorporate most guideline-recommended information when provided with structured prompts. Nevertheless, occasional omissions of specific procedural details highlight the continued importance of clinician review before patient-facing implementation.

Overall, the findings of this study suggest that ChatGPT, Claude, and DeepSeek perform similarly in generating patient education leaflets for cardiac imaging procedures. Although DeepSeek demonstrated better readability and slightly higher information quality, and ChatGPT showed marginally greater guideline adherence, these differences were generally not statistically significant. The results indicate that all three models have considerable potential to assist healthcare professionals in developing patient education materials. However, AI-generated content should complement rather than replace expert review to ensure accuracy, consistency with current guidelines, and suitability for individual healthcare settings [19].

Limitations

This study has several limitations. First, only three cardiac imaging procedures were evaluated, which may limit the generalizability of the findings to other cardiovascular investigations or medical specialties. Second, the study included a relatively small sample of nine AI-generated leaflets, which may have reduced the statistical power to detect differences between the models. Third, the leaflets were assessed by expert reviewers using validated evaluation tools, but patient comprehension, satisfaction, and real-world usability were not evaluated. Readability metrics were used as objective measures of textual complexity and should not be interpreted as direct measures of patient comprehension or health literacy. Future validation studies should assess patient-level comprehension and usability across participants with varying educational and health-literacy backgrounds. Finally, all leaflets were generated using a single standardized prompt and one version of each LLM; alternative prompting strategies or future model updates may produce different results. Despite these limitations, the study provides valuable preliminary insights into the methodological comparability of three AI tools in generating patient education materials.

The small sample size represents an important limitation of the study. With only three generated leaflets per model, the statistical analyses had limited power to detect differences between the LLMs, and both statistically significant and nonsignificant findings should therefore be interpreted cautiously. The descriptive means and standard deviations are presented to characterize the observed outputs rather than as definitive estimates of model-level performance. Larger studies incorporating multiple independently generated outputs across a broader range of cardiac imaging procedures are warranted to provide more robust statistical comparisons.

## Conclusions

ChatGPT, Claude, and DeepSeek were all capable of generating high-quality patient education leaflets for CMR, CTCA, and ICA. Although DeepSeek demonstrated superior readability and ChatGPT achieved the highest guideline adherence, overall performance was comparable across the three models. The principal barrier to clinical implementation was not information quality or guideline adherence, but readability, as all generated leaflets exceeded recommended health literacy standards. These findings suggest that LLMs are best viewed as clinician-assisted drafting tools capable of rapidly producing reliable patient education materials, provided their outputs undergo expert review and readability optimization before use in routine clinical practice.

## Appendices

Google Drive link to LLM prompts and responses: https://drive.google.com/drive/folders/1-4Rr_D8o_PaO0OnIj3NL5cgYgzN-q_Bd?usp=sharing

Google Drive link to the guidelines adherence checklist: https://drive.google.com/drive/folders/1_vyWPb8vLuw7qv-RpL69dQ_v9j0AVuGL?usp=sharing
